# Beyond Self-Reported Adherence and Serology: Stool Gluten Immunogenic Peptides Detection to Assess Gluten Exposure in Children with Celiac Disease on a Gluten-Free Diet

**DOI:** 10.3390/nu18193221

**Published:** 2026-09-29

**Authors:** Fabiana Ziberna, Giorgia Fontana, Chiara Zanchi, Marco Bobbo, Elena Spinelli, Maria Cristina Grimaldi, Fatima Tizi, Grazia Di Leo, Stefano Martelossi, Tarcisio Not, Luigina De Leo

**Affiliations:** 1 Institute for Maternal and Child Health-IRCCS “Burlo Garofolo”, Via dell’Istria 65/1, 34137 Trieste, Italy; fabiana.ziberna@burlo.trieste.it (F.Z.); giorgia.fontana@burlo.trieste.it (G.F.); chiara.zanchi@burlo.trieste.it (C.Z.); mariacristina.grimaldi@burlo.trieste.it (M.C.G.); fatima.tizi@burlo.trieste.it (F.T.); grazia.dileo@burlo.trieste.it (G.D.L.); tarcisio.not@burlo.trieste.it (T.N.); 2 Department of Pediatrics, University of Trieste, 34127 Trieste, Italy; dr@marcobobbo.com; 3 Department of Medical and Surgical Sciences, University of Trieste, 34127 Trieste, Italy; elena.spinelli@burlo.trieste.it; 4 Ca’ Foncello Hospital, 31100 Treviso, Italy; stefano.martelossi@aulss2.veneto.it

**Keywords:** gluten-free diet, celiac disease, gluten immunogenic peptides, Biagi questionnaire

## Abstract

**Background/Objectives:** A lifelong gluten-free diet (GFD) is the only treatment for celiac disease (CD). However, current methods for monitoring GFD adherence do not accurately assess gluten exposure. Stool detection of gluten immunogenic peptides (GIP) may provide an objective tool for detecting recent gluten exposure. The aim of this study was to evaluate stool GIP detection, compared with serology and the validated Biagi dietary questionnaire, for monitoring GFD adherence in children with CD. **Methods:** In this prospective multicenter study, pediatric patients with CD on a GFD for at least 1 year were enrolled. Serum and stool samples were collected on the same day to assess serum anti-transglutaminase (TTG) and anti-endomysium (EMA) antibodies and stool GIP. Patients also completed the Biagi dietary questionnaire. **Results:** Forty-one pediatric patients with CD on a GFD for a median of 6.5 years were enrolled. Serum TTG and/or EMA were positive in 8/41 patients (19.5%), Biagi questionnaire identified 8/41 patients (19.5%) as non-adherent, whereas stool GIP was positive in 17/41 (41.5%). Combining serology with the dietary questionnaire increased the proportion of non-adherent patients to 34%. Adding stool GIP detection increased this proportion to 51% and 46% when combined with serology or the dietary questionnaire, respectively. Overall, 23/41 patients (56%) showed evidence of gluten exposure when positivity in at least one of the three methods was considered. **Conclusions:** A high rate of recent gluten exposure was detected in children with CD on a long-term GFD. Stool GIP detection may provide complementary information to conventional methods for monitoring GFD adherence and may help identify patients requiring further dietary assessment or intervention.

## 1. Introduction

Celiac disease (CD) is a chronic immune-mediated enteropathy triggered and maintained by exposure to gluten in genetically susceptible individuals. Currently, the only available treatment for CD is a strict lifelong gluten-free diet (GFD). Adherence to a GFD usually leads to complete mucosal recovery, resolution of symptoms, and improved quality of life, while reducing the risk of long-term complications [1]. However, a GFD is burdensome and strict dietary adherence may be challenging both for children and adults [2]. Despite their best efforts, 30% to 60% of patients with CD are exposed to gluten due to cross-contamination, social pressure when eating out, the limited availability and high cost of gluten-free products, and the absence or nonspecific nature of symptoms following dietary transgressions [3,4]. Therefore, monitoring adherence to GFD in patients with CD is essential for disease management and recommended by current guidelines [5].

The assessment of adherence to GFD includes mainly dietary questionnaires and serial measurement of CD-related antibodies. Unfortunately, these procedures have limitations: they lack of sensitivity for detecting low-level or occasional gluten exposure [6], normalization of CD-related antibodies does not necessarily correlate with the intestinal recovery [7], and dietary questionnaires rely on patients’ subjective reports.

Over the last decade, efforts have been addressed to find objective and reliable tools to detect dietary lapses for monitoring GFD. These tools are based on ELISA and lateral flow immunoassays detecting gluten immunogenic peptides (GIP) in stool or urine with high specificity and sensitivity [8,9,10,11,12]. GIP are resistant to gastric and intestinal degradation and represent a promising marker of gluten consumption. GIP can be detected in stool within 3 days after minimal ingestion [8] and in urine within several hours of ingestion [9]. However, the role of GIP testing during follow-up of children on a GFD is under debate.

In this study, we hypothesized that stool GIP testing would provide complementary information to conventional methods for assessing adherence to a GFD by identifying recent gluten exposure that may not be detected by dietary questionnaires or CD-specific serology. Therefore, the aim of this study was to evaluate stool GIP measurement as a marker of GFD adherence in pediatric patients with CD on a GFD and to compare the results with two conventional methods for assessing GFD adherence: measurement of serum CD-related antibodies and a validated dietary questionnaire.

## 2. Materials and Methods

### 2.1. Patients and Study Design

This was a multicenter prospective study conducted at the tertiary referral center IRCCS Burlo Garofolo (in Trieste) and at Ca’ Foncello Hospital (in Treviso) between January 2019 and January 2020. Patients were eligible if they had been diagnosed with CD between 1 and 18 years of age and had been on a GFD for at least 1 year. Children with other concomitant gastrointestinal disorders (e.g., inflammatory bowel disease, eosinophilic esophagitis) were excluded. Patients with CD who fulfilled the inclusion criteria were invited during their scheduled medical visits to participate in the study. They were asked to provide one stool sample together with a blood sample and their GFD adherence was assessed by a questionnaire. Informed consent was obtained from parents as well as from the patients. The study was approved by the Institutional Review Board of Institute for Maternal and Child Health-IRCCS “Burlo Garofolo”, Trieste, Italy (n° 7/2019, date of approval 27 August 2019).

### 2.2. Samples Collection

Subjects were instructed to collect and keep at −20 °C a stool sample. Specimens were delivered and kept at −20 °C until processing.

Blood sample was collected within 24 h of stool collection.

### 2.3. Evaluation of GFD Compliance

#### 2.3.1. CD-Related Antibodies Determination

Serum anti-tissue transglutaminase antibodies (TTG) were measured using commercial ELISA assay (Eurospital, Trieste, Italy) and values higher than 9 U/mL for IgA were considered positive. Serum IgA anti-endomysium antibodies (EMA) were investigated by an indirect immunofluorescence method using sections of monkey esophagus as substrate, following the manufacturer’s instructions (Eurospital kit Antiendomysium 96, Trieste, Italy).

In patients with selective IgA deficiency (total IgA < 5 mg/dL), serum samples were investigated for IgG TTG (positive ≥ 20 U/mL) and IgG1 EMA.

#### 2.3.2. GIP Test

Stool GIP concentration was measured by an ELISA assay (iVYDAL In Vitro Diagnostics iVYLISA GIP Stool kit, Biomedal, Seville, Spain) according to the manufacturer’s instructions. Briefly, GIP were extracted from the stool by incubating the sample with an extraction solution at 50 °C for 60 min. The extracted samples were analyzed by ELISA, and GIP concentrations were expressed as ng/g of feces. Values >78 ng GIP/g feces were considered positive.

#### 2.3.3. Dietary Assessment

The Biagi score questionnaire was used to evaluate GFD compliance [13]. The score is calculated on just four simple questions. The answers lead to different final scores which stratify the patients in three different levels: patients with a score of 0 or 1, who do not follow the GFD; patients with a score of 2, who follow a GFD characterized by important errors that should be corrected; patients with a score of 3 or 4, who follow a strict GFD.

### 2.4. Statistical Analyses

Statistical analyses were performed using GraphPad Prism version. 5.3 (GraphPad Software, San Diego, CA, USA). Data were reported as absolute and relative frequency (nr, %) for the categorical variables, as median ± standard deviation (SD) for continuous variables. The chi-squared test (Fisher) or Mann–Whitney U as appropriate were used to analyze the differences between groups for quantitative variables. Correlation was measured with Spearman’s correlation coefficient. The analyzed parameters were sex, age at diagnosis and at visit, and duration of the GFD. For all analyses, a *p*-value < 0.05 was considered statistically significant. Cohen’s kappa was used to evaluate the agreement between methods. The scale to determine the degree of concordance was the following: ≤0 “no agreement”; 0.01–0.20 “weak agreement”; 0.21–0.40 “fair agreement”; 0.41–0.60 “moderate agreement”; 0.61–0.80 “substantial agreement”; 0.81–0.99 “almost perfect agreement”; 1.00 “perfect agreement”.

## 3. Results

### 3.1. Patients

**Figure 1:**
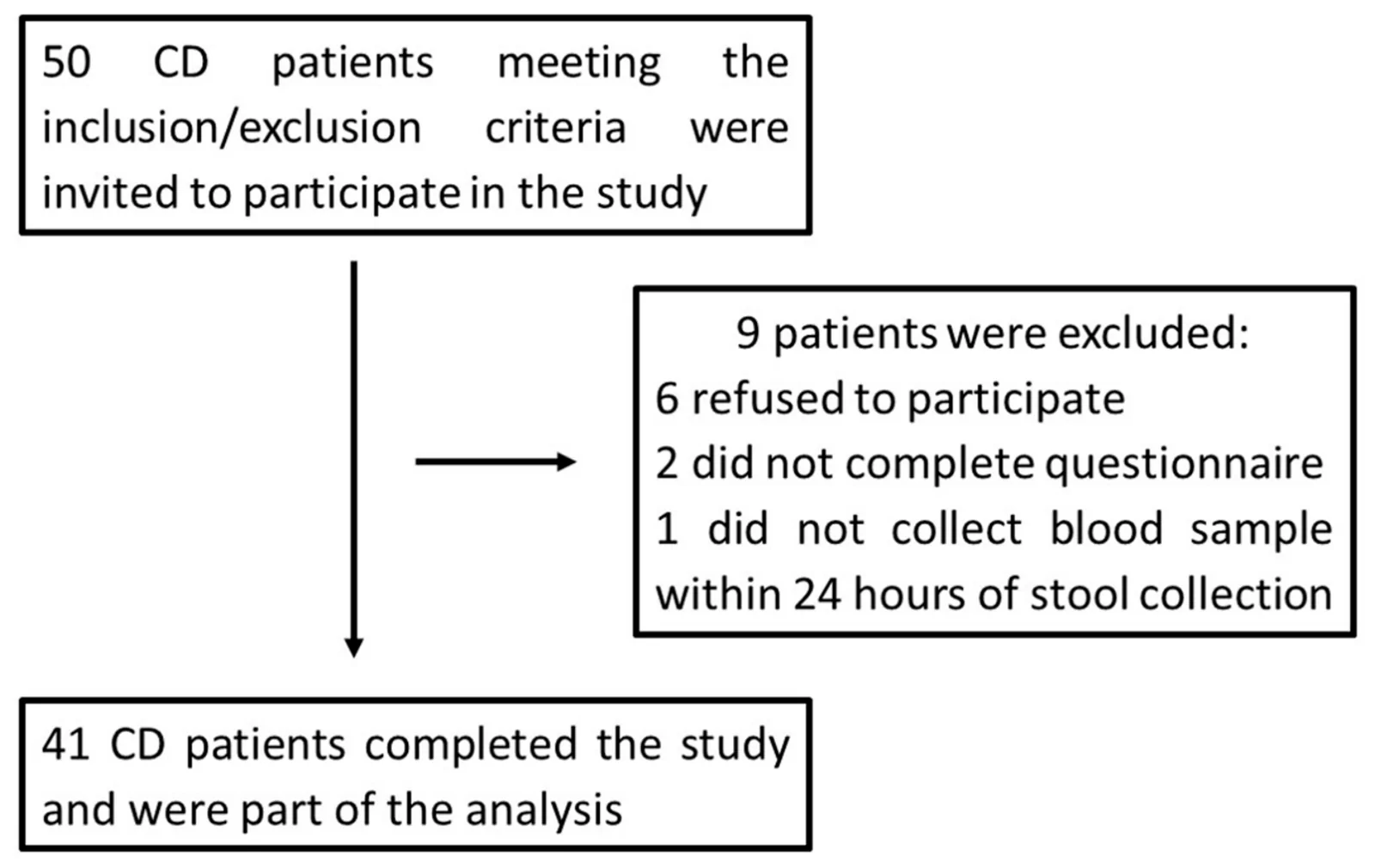
Participant flowchart. Flow of participants from eligibility assessment through final analysis. Fifty patients that met the inclusion criteria were enrolled in the study. Nine patients were excluded. A total of 41 patients completed the study and were considered for the final analysis. Abbreviations: CD = celiac disease.

Figure 1 represents the flowchart of pediatric patients recruited in the study. A total of 41 children with CD (29 females, 12 males; median age 14 years) on a GFD completed the study and were enrolled from two Italian hospitals, and both stool and serum samples were collected along with dietary assessment.

The median age at diagnosis of CD was 7 years (range: 1–15 ys), and patients had been on a GFD for a median duration of 6.5 years (range: 1–17 ys). Twenty-seven of 41 (66%) patients presented with symptoms at diagnosis. Of these, 18 exhibited gastrointestinal manifestations, such as diarrhea, meteorism, and abdominal pain. The remaining nine patients primarily showed extraintestinal symptoms, including asthenia, loss of appetite and failure to thrive. Fourteen of 41 (34%) patients were asymptomatic and were tested for CD as first-degree relatives of affected individuals. Two patients (5%) presented with IgA deficiency. The demographic characteristics of the study population are summarized in Table 1.

### 3.2. Assessment of GFD Adherence Using Serological CD-Related Antibodies, Stool GIP Detection, and Qualitative Dietary Assessment

The main clinical and laboratory characteristics of the patients are summarized in Table 2.

**Figure 2:**
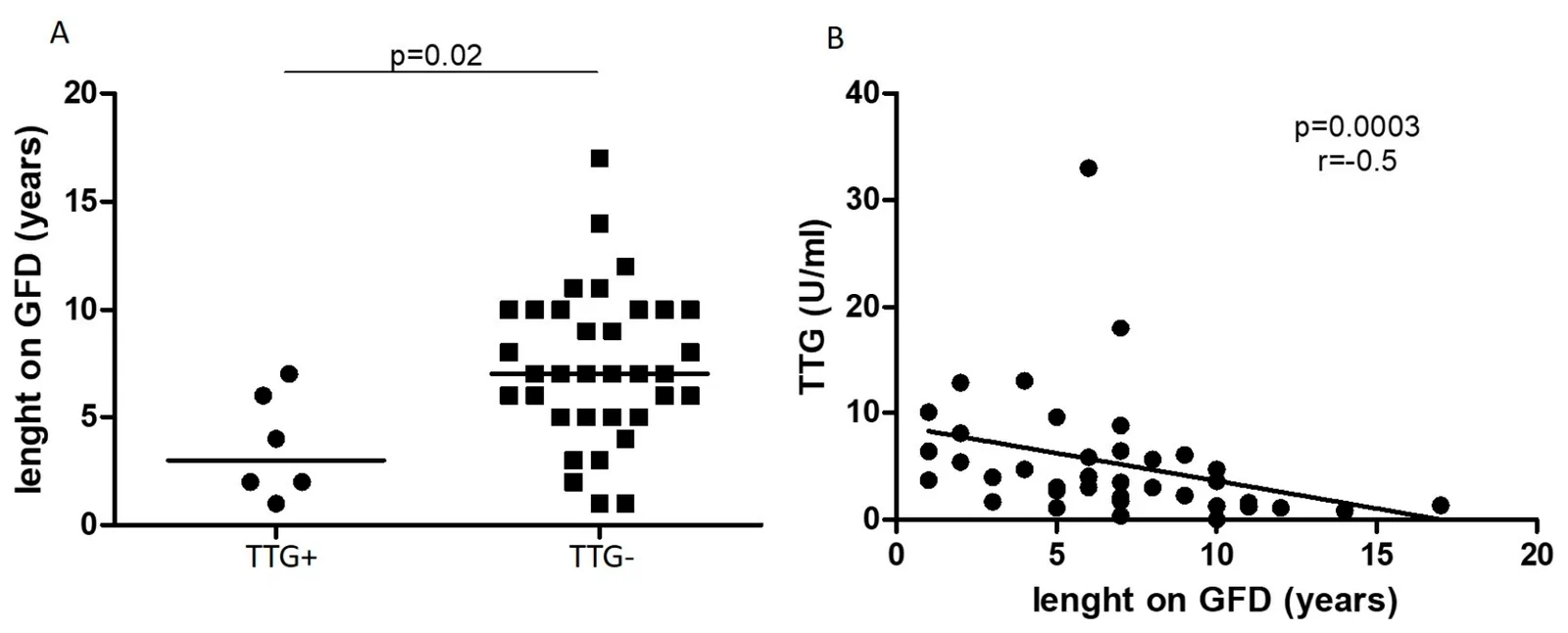
Association and correlation between serum TTG and GFD duration. (**A**) The duration of the GFD was significantly shorter in TTG-positive (TTG+) than in TTG-negative (TTG−) patients (*p* = 0.02), as assessed by the Mann–Whitney U test. (**B**) TTG levels were significantly higher in patients with a shorter GFD duration, as assessed by Spearman’s correlation coefficient (*p* = 0.0003, r = −0.5). Abbreviations: GFD = gluten-free diet, TTG = anti-transglutaminase antibodies.

Eight of 41 (19.5%) patients tested positive for TTG (11.5 U/mL ± 2.4 for IgA; 25.5 U/mL ± 10.6 for IgG in the two patients with IgA deficiency) and/or EMA: 3/8 were positive for TTG only, 2/8 were positive for EMA only and 3/8 for both TTG and EMA. Six of these eight (75%) patients were symptomatic patients at diagnosis. A significant association (*p* = 0.02) and a significant negative correlation (r = −0.5, *p* = 0.0003) were observed only between serum TTG levels and the duration of the GFD, indicating that longer adherence to the GFD was associated with lower serum TTG levels (Figure 2A,B).

Positive stool GIP (242.5 ng GIP/g feces ± 320.7) were detected in 17 of 41 (41.5%) patients, of whom 15 (88%) were symptomatic at diagnosis. No associations or correlations were found between stool GIP levels and the parameters analyzed.

Assessment of adherence to the GFD using the Biagi questionnaire showed good adherence (Biagi score 3–4) in 33/41 patients (80.5%) and poor adherence (Biagi score 0–1) in 8/41 patients (19.5%). Notably, two of eight patients (25%) with poor adherence did not follow a GFD at all (Biagi score 0). Seven of these eight patients reporting poor adherence to the GFD were symptomatic at diagnosis. None of the patients had a Biagi score 2.

No significant associations or correlations were found between the Biagi score and the parameters analyzed.

### 3.3. Comparative and Integrative Analysis of Serological CD-Related Antibodies, Stool GIP Detection and Qualitative Assessment GFD Adherence

**Figure 3:**
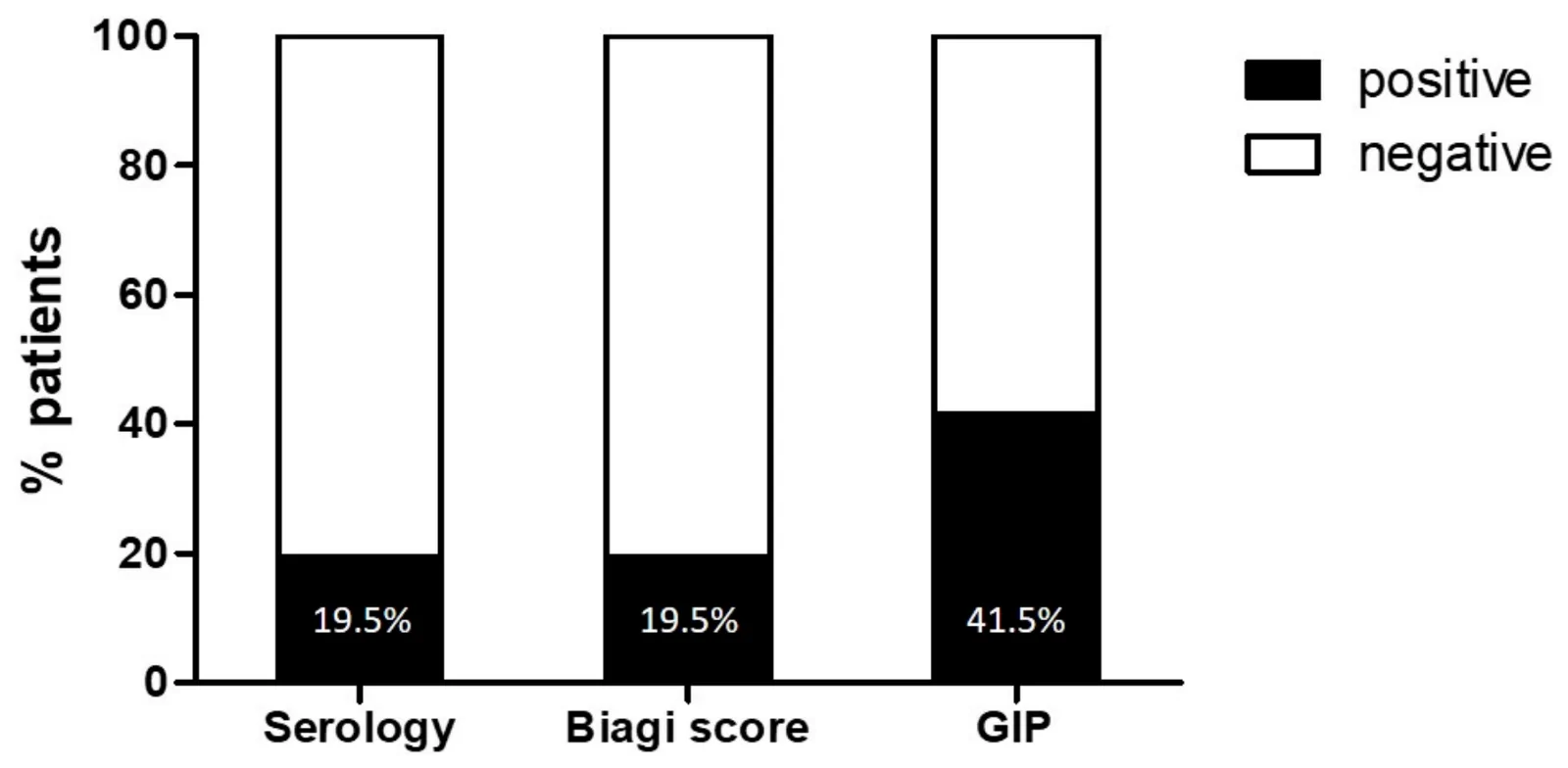
Assessment of GFD adherence in the study population using three methods: serological tests, qualitative dietary questionnaire (Biagi score) and stool GIP detection. The percentage of patients with evidence of GFD non-adherence was 19.5% by serological celiac disease-related antibodies (TTG and/or EMA) assessment, 19.5% by the Biagi score questionnaire, and 41.5% by stool GIP detection. Abbreviations: EMA = anti-endomysium antibodies, GFD = gluten-free diet, GIP = gluten immunogenic peptides, TTG = anti-transglutaminase antibodies.

Both serological testing for CD-related antibodies and qualitative assessment of GFD adherence identified 19.5% of patients as non-adherent, whereas stool GIP analysis identified 41.5% of patients as having evidence of gluten exposure (Figure 3).

**Figure 4:**
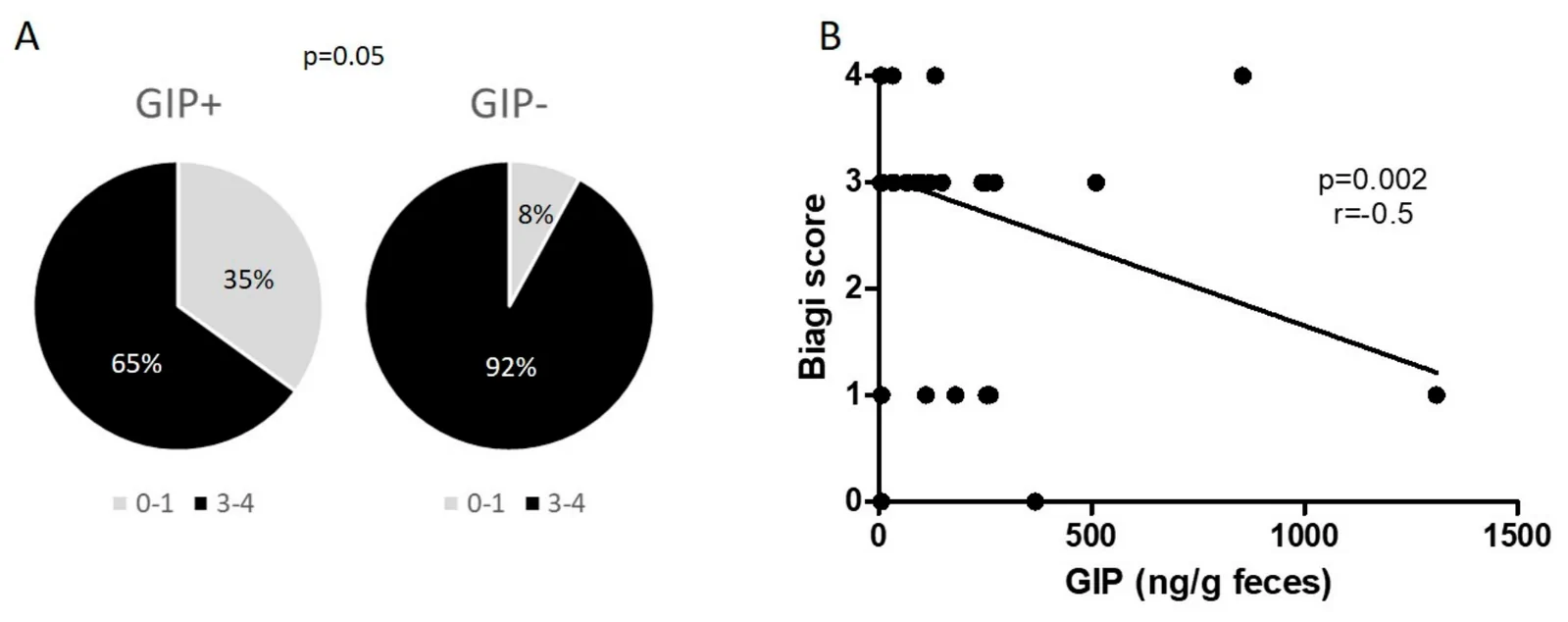
Association and correlation between Biagi score questionnaire and stool GIP detection. (**A**) The two pie graphs show the percentage of patients with poor GFD adherence (0–1) and good GFD adherence (3–4) among GIP-positive (GIP+) and GIP-negative (GIP−) patients, based on Biagi score questionnaire. The difference was statistically significant (*p* = 0.05), as assessed by the Mann–Whitney U test. (**B**) Biagi score was significantly higher in patients with low stool GIP levels, as assessed by Spearman’s correlation coefficient (*p* = 0.002, r = −0.5). Abbreviations: GFD = gluten-free diet, GIP = gluten immunogenic peptides.

The stool GIP test showed weak agreement with CD-related antibody determination (κ = 0.07) and fair agreement with the Biagi score (κ = 0.3). Likewise, only weak agreement was observed between CD-related antibody determination and the Biagi score (κ = 0.07). An association and correlation were observed only between the Biagi score and stool GIP test. Among GIP-positive patients, 11/17 (65%) reported good adherence to the GFD, compared with 22/24 (92%) of GIP-negative patients (*p* = 0.05), suggesting a discrepancy between self-reported adherence and objectively detected gluten exposure. The Biagi score was inversely correlated with stool GIP concentrations, with higher Biagi scores associated with lower GIP levels (r = −0.50, *p* = 0.002) (Figure 4A,B).

**Figure 5:**
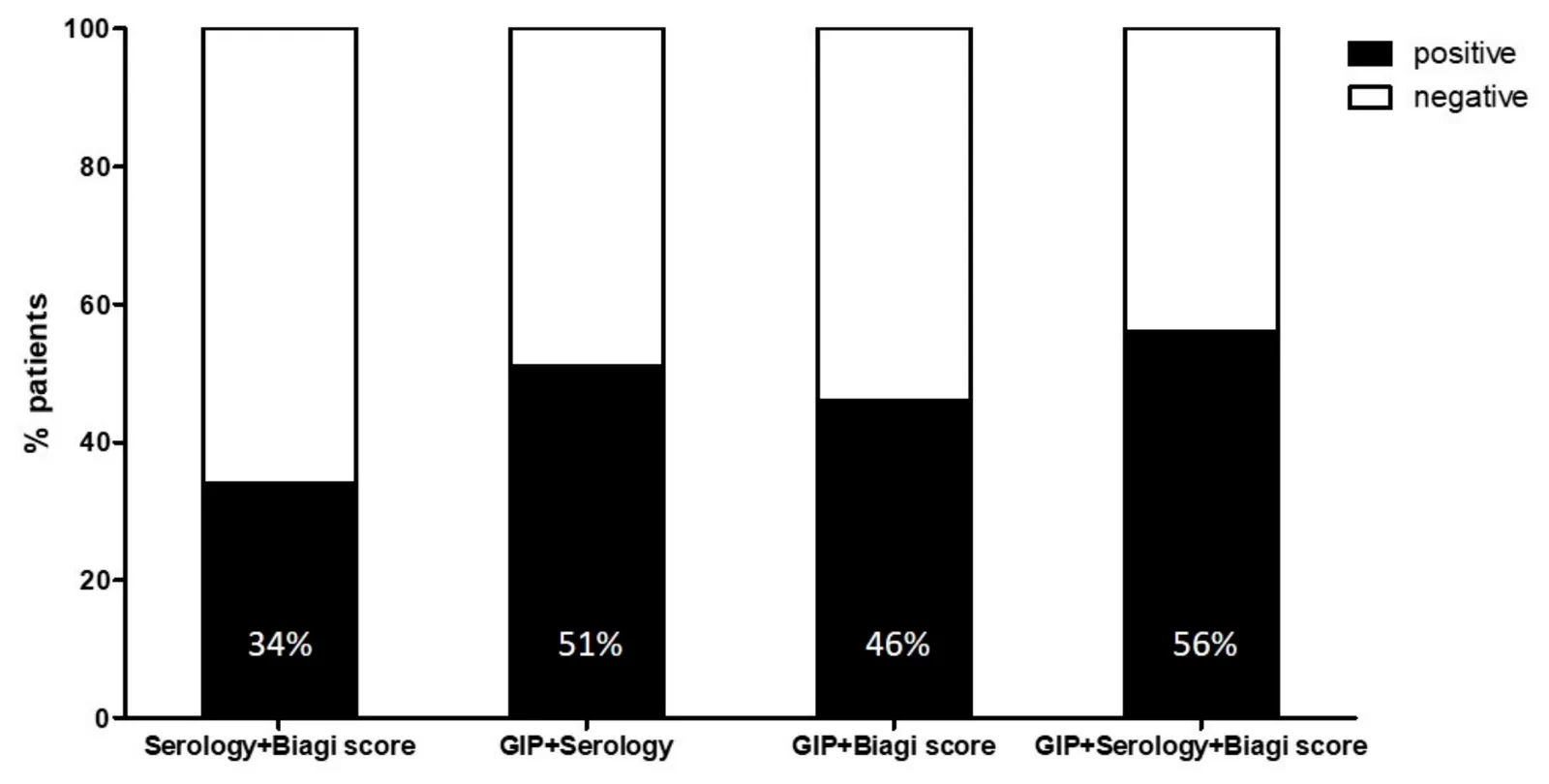
Assessment of GFD adherence in the study population using different combinations of three methods: serological tests (serum TTG and/or EMA), qualitative dietary questionnaire (Biagi score) and stool GIP detection. Evidence of GFD non-adherence was found in 34% of patients based on the combination of serological assessment and the Biagi score questionnaire, 51% based on serological assessment and stool GIP detection, 46% based on stool GIP detection and the Biagi score questionnaire, and 56% based on all three methods. Abbreviations: EMA = anti-endomysium antibodies, GFD = gluten-free diet, GIP = gluten immunogenic peptides, TTG = anti-transglutaminase antibodies.

The combination of the two conventional methods for assessing adherence to the GFD in patients with CD, namely CD-related antibody testing and the dietary questionnaire, increased the proportion of patients identified as non-adherent from 19.5% when each method was considered individually to 34% when positivity in at least one of the two methods was considered. When stool GIP detection was combined with CD-related antibody testing or the dietary questionnaire, the proportions of patients identified as non-adherent increased to 51% and 46%, respectively. Finally, when positivity in at least one of the three analyzed methods (CD-related antibody testing, Biagi score, and stool GIP) was considered, 23 of 41 patients (56%) showed evidence of either suboptimal adherence to the GFD or recent gluten exposure (Figure 5).

## 4. Discussion

Strict adherence to a GFD is the current recommended strategy in CD patients to ensure symptom resolution, mucosal healing, and prevention of complications. However, maintaining strict compliance is challenging. Therefore, accurate assessment of adherence is of paramount importance for optimal disease management. Strategies for monitoring dietary adherence mainly rely on patient-reported questionnaires assessing gluten intake and on laboratory tools measuring celiac serology, both of which have limited sensitivity and/or specificity. The detection of stool GIP seems to represent an effective marker to detect recent gluten intake and to assist clinicians in assessment of GFD compliance.

This Italian multicenter study included pediatric patients with CD who had been on a GFD for a long median duration of 6.5 years. In this cohort, stool GIP were detected in 41.5% of patients, despite 80.5% reporting good adherence to the GFD and 80.5% testing negative for serum TTG and EMA. These findings highlight, on the one hand, the discrepancy between patients’ perceived adherence to a strict GFD and actual dietary gluten exposure. This suggests that inadvertent gluten ingestion is common even among patients who report good dietary adherence, consistent with previous studies [8,10,11]. On the other hand, our results further support the limited value of conventional CD serology for monitoring adherence to a GFD, as negative TTG and EMA results do not reliably exclude ongoing gluten exposure. The sensitivity and specificity of serum TTG and EMA for assessing adherence to a GFD are limited [7]. Although positive CD-specific serology generally indicates ongoing gluten exposure, negative serological results do not reliably exclude chronic or intermittent dietary lapses [12]. In our study, there was a significant negative correlation between serum TTG levels and GFD duration. This finding is consistent with the concept that antibody levels reflect the underlying immune response and may take time to decline and become negative, making serological markers potentially unreliable for detecting small or intermittent gluten exposure. Consistent with a recent study [14], our results also showed no relationship between serum TTG levels and stool GIP results. Some patients with negative serological markers had positive stool GIP results, suggesting recent gluten exposure prior to testing.

It remains to be established whether stool GIP testing is an appropriate tool for assessing adherence to a GFD or whether the high sensitivity of this test may be misleading in clinical practice. Indeed, the clinical significance of occasional minimal gluten exposure remains a matter of debate, as the maximum amount of gluten that can be safely tolerated by patients with CD has not yet been clearly established. Based on previous studies, a threshold of 10 mg of gluten per day is generally considered safe for most patients with CD [15].

A significant inverse correlation was observed between the Biagi score and stool GIP levels (r = −0.50, *p* = 0.002), indicating that higher adherence to the GFD was associated with lower stool GIP levels. However, stool GIP were detectable in 41.5% of patients, whereas only 19.5% reported poor adherence to the GFD, suggesting a discrepancy between self-reported dietary adherence and objectively detected gluten exposure. These findings suggest that objective GIP testing may provide additional information on gluten exposure not captured by self-reported dietary assessment, even among patients reporting good adherence to the GFD.

When considered individually, conventional methods identified 19.5% of patients as non-compliant, whereas their combination increased this proportion to 34%. Importantly, the inclusion of stool GIP testing further increased the proportion of patients with evidence of gluten exposure, with 23/41 (56%) patients showing positivity in at least one of the three assessments. These findings highlight the complementary nature of the different approaches used to assess adherence to the GFD, as evinced by the weak agreement observed between the tests. Different tools identify different subsets of patients: self-reported adherence is associated with objective gluten exposure, but does not fully capture it; stool GIP positivity reflects recent gluten exposure but does not necessarily distinguish between intentional and unintentional gluten ingestion; and positive CD serology indicates a gluten-related immune response activation. Given that strict adherence to the GFD is essential for patients with CD, these findings suggest that reliance on a single assessment method may underestimate non-adherence. The higher detection rate observed when objective GIP testing was combined with conventional approaches supports the potential value of a multimodal assessment of adherence, particularly in patients who report good dietary adherence but may nevertheless have evidence of gluten exposure. In patients with positive stool GIP results despite negative serology and self-reported good adherence to the GFD, clinicians may consider repeating GIP testing on subsequent stool samples and asking patients to keep a detailed food diary during the week preceding stool collection. This approach may help identify potential sources of inadvertent or unintentional gluten exposure that would otherwise remain unrecognized.

Some limitations of this study should be acknowledged. First, the number of patients included was relatively small. Second, each patient provided only a single stool sample, and relying on a single GIP measurement may have influenced the results. Since stool GIP testing provides information on recent gluten exposure, occasional or repeated gluten ingestion occurring several days before stool collection may not have been detected, potentially leading to an underestimation of both incidental and non-incidental deviations from the GFD. In cases of suspected or uncertain adherence, repeated GIP measurements may therefore be useful to increase the likelihood of detecting gluten exposure. Indeed, Stefanolo et al. [16] reported that testing for GIP three times per week detected gluten exposure in 89% of cases. Moreover, patients who were aware that their adherence to the GFD would be assessed may have temporarily increased their adherence in the days preceding the study visit. Consequently, the true prevalence of suboptimal adherence may be higher in real-world clinical practice.

## 5. Conclusions

In conclusion, this study demonstrates a high rate of recent gluten exposure among patients with CD who had been following a long-term GFD and highlighted the potential added value of stool GIP detection alongside conventional methods for monitoring GFD adherence. Stool GIP testing may play an important role in the follow-up of patients with CD by identifying gluten exposure and helping clinicians determine when further assessment or intervention by a clinician or dietitian may be warranted. However, further studies are needed to clarify the clinical utility of stool GIP testing for assessing adherence to a GFD and to determine how to integrate this tool into clinical practice.

## Figures and Tables

**Table 1:** Demographic characteristics of study population.

Characteristics	Overall	Symptomatic	Asymptomatic	*p*-Value
Cases *n* (%)	41	27 (66%)	14 (34%)	
Sex *n* (%)	F = 29 (71%)	F = 20 (74%)	F = 9 (64%)	0.72
Median age at diagnosis (range) in years	7 (1–15)	9 (1–15)	7 (2–11)	0.45
Median age at visit (range) in years *	14 (8–24)	15 (10–21)	12 (8–24)	0.15
Median time on GFD (range) in years	6.5 (1–17)	7 (1–14)	7 (1–17)	0.8

Values are reported as *n* (%) for categorical variables and as median ± SD for continuous variables. Study population included 41 pediatric patients, classified as symptomatic or asymptomatic according to the presence or absence of symptoms at diagnosis. The analyzed parameters were sex, age at diagnosis and at visit, and duration of the GFD. For all analyses, a *p*-value < 0.05 was considered statistically significant. Abbreviations: F = female, GFD = gluten-free diet, SD = standard deviation. * Five patients were diagnosed with celiac disease during childhood and were evaluated as young adults after a prolonged period on a gluten-free diet.

**Table 2:** Clinical and laboratory characteristics of study population.

Characteristics	Patients (*n* = 41) *n* (%)
**Serology**	
TTG-positive	6 (14.6%)
EMA-positive	5 (12%)
Serology positivity **^1^**	8 (19.5%)
**GIP stool**	
GIP stool-positive	17 (41.5%)
Median titers of GIP-positive (SD) in ng/g feces	242.5 (320.7)
**Rate of Biagi score 0–1**	8 (19.5%)

^1^ TTG- and/or EMA-positive. The main clinical and laboratory characteristics of the patients are summarized here. Values are reported as *n* (%) for categorical variables and as median ± SD for continuous variables. Abbreviations: EMA = anti-endomysium antibodies, GIP = gluten immunogenic peptides, SD = standard deviation, TTG = anti-transglutaminase antibodies.

## Data Availability

The original contributions presented in this study are included in the article. Further inquiries can be directed to the corresponding author.

## Abbreviations

The following abbreviations are used in this manuscript:

CD	celiac disease
EMA	anti-endomysium antibodies
GFD	gluten-free diet
GIP	gluten immunogenic peptides
TTG	anti-transglutaminase antibodies
